# CD14 and ALPK1 Affect Expression of Tight Junction Components and Proinflammatory Mediators upon Bacterial Stimulation in a Colonic 3D Organoid Model

**DOI:** 10.1155/2020/4069354

**Published:** 2020-02-01

**Authors:** Pascal Brooks, Talke zur Bruegge, Erin C. Boyle, Stefan Kalies, Santiago Nahuel Villarreal, Andrea Liese, André Bleich, Manuela Buettner

**Affiliations:** ^1^Institute for Laboratory Animal Science, Hannover Medical School, Carl-Neuberg-Str. 1, 30625 Hannover, Germany; ^2^Institute of Quantum Optics, Leibniz University Hannover, Hannover, Germany; ^3^Lower Saxony Center for Biomedical Engineering, Implant Research and Development (NIFE), Hannover, Germany

## Abstract

*Cd14* and *Alpk1* both encode pathogen recognition receptors and are known candidate genes for affecting severity in inflammatory bowel diseases. CD14 acts as a coreceptor for bacterial lipopolysaccharide (LPS), while ALPK1 senses ADP-D-glycero-beta-D-manno-heptose, a metabolic intermediate of LPS biosynthesis. Intestinal barrier integrity can be influenced by CD14, whereas to date, the role of ALPK1 in maintaining barrier function remains unknown. We used colon-derived 3D organoids, first characterised for growth, proliferation, stem cell markers, and expression of tight junction (TJ) components using qPCR and immunohistochemistry. They showed characteristic crypt stem cells, apical shedding of dead cells, and TJ formation. Afterwards, organoids of different genotypes (WT, *Il10*^−/−^, *Cd14*^−/−^, and *Alpk1*^−/−^) were then stimulated with either LPS or *Escherichia coli* Nissle 1917 (*Ec*N). Gene expression and protein levels of cytokines and TJ components were analysed. WT organoids increased expression of *Tnfα* and tight junction components. *Cd14*^−/−^ organoids expressed significantly less *Tnfα* and *Ocln* after LPS stimulation than WT organoids but reacted similarly to WT organoids after *Ec*N stimulation. In contrast, compared to WT, *Alpk1*^−/−^ organoids showed decreased expression of different TJ and cytokine genes in response to *Ec*N but not LPS. However, Western blotting revealed an effect of ALPK1 on TJ protein levels. These findings demonstrate that *Cd14*, but not *Alpk1*, alters the response to LPS stimulation in colonic epithelial cells, whereas *Alpk1* is involved in the response upon bacterial challenge.

## 1. Introduction

Inflammatory bowel disease (IBD) onset involves the interplay between microbiota, environmental conditions, genetic factors, and a disrupted intestinal barrier [1]. Several mouse models of IBD have been established [2] including the well-studied *Il10* (interleukin-10) deficiency model. This model is characterised by a dysregulated immune response to enteric microflora leading to the onset of colitis through a disrupted barrier due to increased levels of interferon gamma and tumor necrosis factor alpha (TNF*α*) [3]. Using the *Il10^−/−^* mouse model and quantitative trait locus (QTL) analyses, several studies have identified genetic loci associated with susceptibility to IBD. These studies revealed ten *cytokine deficiency-induced colitis susceptibility* (*Cdcs*) loci and candidate genes with a potential influence on colitis onset [4–9]. One of these genes is *Cd14* (Cluster of differentiation 14) in the *Cdcs6* locus located on chromosome 18 [7]. CD14 acts as a coreceptor of Toll-like receptor (TLR) 4 and is directly involved in the detection of lipopolysaccharide (LPS) and activation of NF-*κ*B [10]. In previous studies, our group demonstrated that CD14 has a protective function on barrier integrity in a dextran sodium sulfate- (DSS-) induced acute colitis mouse model [11]. We also showed that in wild-type (WT) but not in *Cd14*^−/−^ mice, monoassociation with *Escherichia coli* Nissle 1917 (*Ec*N) leads to increased expression of tight junction (TJ) components, associated with protection against bacterial translocation [12].

Another candidate gene revealed by these QTL studies is *Alpk1* (alpha-protein kinase 1) which is located on chromosome 3 in the *Cdcs1* locus [4, 5]. Knockout of the *Alpk1* gene in mice was recently shown to lead to severe colitis when infected with *Helicobacter hepaticus* [13]. ALPK1 functions as a pattern recognition receptor for adenosine diphosphate-heptose (ADP-Hep), a precursor of LPS [14]. In human epithelial cells, ADP-Hep of Gram-negative bacteria activates NF-*κ*B-dependent inflammatory interleukin-8 (IL-8) secretion via an ALPK1-TIFA-TRAF6 axis [15]. As mice lack a functional *Il-8* gene, the chemokines CXCL1, CXCL2, and CXCL5 are regarded as functional homologues [16].

To study the specific impact of *Il10*, *Cd14*, and *Alpk1* on intestinal epithelial cell (IEC) response to bacterial stimulation, we used colonic organoid cultures derived from isolated intestinal stem cells (ISCs) [17]. These ISCs can differentiate into all colonic epithelial lineages including colonocytes, goblet cells, and several enteroendocrine cell types [18]. In the present study, colonic organoids of different knockout mouse strains were stimulated with LPS or *Ec*N and the expression of proinflammatory mediators and tight junction components was determined. Here, we report that IEC *Cd14* and *Alpk1* both affect cytokine and TJ component expression upon bacterial challenge.

## 2. Materials and Methods

### 2.1. Mice

This study was conducted in accordance with German animal protection law and with the European Directive 2010/63/EU. All experiments were approved by the Local Institutional Animal Care (File: 2015/78). Phenotypically healthy female and male, 8 to 18 weeks old, C57BL/6J (WT), C57BL/6J.129P2-*Il10^tm1Cgn^* (B6-*Il10*^−/−^), C57BL/6J.129S1-*Cd14^tm1Smg^* (B6-*Cd14*^−/−^), C57BL/6N (*Alpk1^+/+^*), C57BL/6N-*Alpk1^em2Wtsi^* (*Alpk1*^−/−^), and B6.129P2-*Lgr5^tm1(cre/ERT2)Cle^*/J (*Lgr5-eGFP^+/-^*) mice were obtained from the Central Animal Facility (Hannover Medical School, Hannover, Germany). All mice were housed under specific pathogen-free conditions in individually ventilated cages.

### 2.2. Preparation of Organoids

The protocol was based on Mahe et al. [18]. Mice were sacrificed by CO_2_ inhalation followed by cervical dislocation at an age of 10-14 weeks. The entire colon was flushed and washed with sterile, ice-cold Dulbecco's phosphate-buffered saline (DPBS) in a sterile environment. All further steps were performed on ice. Tissues were opened lengthwise and cut into small pieces, washed several times with DPBS, transferred into crypt chelating buffer (CCB) (DPBS containing 0.5 M EDTA), and incubated for 30 minutes on a rocking platform. After washing with CCB, colon pieces were transferred into dissociation buffer (DB) (DPBS containing 54.9 mM sorbitol and 43.4 mM sucrose) and mixed thoroughly. Cell suspensions were filtered and centrifuged, and cells were resuspended in an appropriate volume of Matrigel® membrane matrix (Corning™, New York, USA) (Matrigel®) to yield approximately 1000 crypts/ml. Fifty *μ*l of the cell-Matrigel® mixture was carefully pipetted into the centre of each well of a 24-well plate, incubated for 30 minutes at 37°C for solidification, and overlaid with 500 *μ*l organoid growth media (DMEM, high glucose, GlutaMAX™, pyruvate (Thermo Fisher Scientific, Massachusetts, USA)) supplemented with 50% L-WRN-supernatant (ATCC® CRL3276™ in DMEM, high glucose, GlutaMAX™, pyruvate plus 10% fetal calf serum), 1x N2 (Invitrogen, Carlsbad, USA), 1x B27 (Invitrogen, Carlsbad, USA), 50 ng/*μ*l recombinant mouse epidermal growth factor (Sigma-Aldrich, St. Louis, USA), 10 *μ*M Y-27632 (Tocris, Bristol, UK), and 1x Cellshield (Biochrom, Berlin, Germany). Organoids were cultured at 37°C with 5% CO_2_. Media was exchanged every 3-4 days.

### 2.3. Organoid Kinetics

Matrigel® was dissolved using ice-cold DPBS, and crypts were separated by drawing the suspension up once through a syringe using a 27G 1/2^″^ needle. Cells were washed and seeded into fresh Matrigel®, which was allowed to solidify for 30 minutes at 37°C, and afterwards overlaid with 500 *μ*l organoid growth media. Pictures were taken on days 1, 3, 5, 7, 9, 11, and 13 after seeding. At each time point, three samples were obtained for RNA isolation by pooling two wells of a 24-well plate for each sample.

### 2.4. Hematoxylin and Eosin (H&E) Staining

Matrigel®-embedded organoids were embedded in 4% agarose, fixed with 4% formalin overnight, dehydrated, and cut into 4 *μ*m sections. A standard H&E staining was performed.

### 2.5. Immunofluorescence Staining

Matrigel®-embedded organoids were whole mount stained. Therefore, they were fixed for 1 h with 4% formalin. Autofluorescence was quenched via incubation with NH_4_Cl for 1 h. Permeabilisation and blocking were performed for 1.5 h with 0.5% Triton-X100 and 10% horse serum in DPBS. Organoids were stained with rat anti-EpCAM/CD326-FITC, rabbit anti-occludin, rabbit anti-tight junction protein 1 (TJP1; also known as zonula occludens 1, ZO-1), rabbit anti-claudin 8 (all Invitrogen, Carlsbad, USA), or rabbit anti-Ki67 (Abcam, Cambridge, UK). Unconjugated antibodies were visualised in a second step by a goat anti-rabbit DyLight 488 antibody (BioLegend, San Diego, USA). Primary and secondary antibodies were diluted in 0.1% Triton-X100, 10% horse serum in DPBS, and incubated overnight at 4°C. Images were acquired using a confocal TCS SP5 microscope system (Leica, Wetzlar, Germany).

### 2.6. TUNEL Staining

Preparation of Matrigel®-embedded organoids was performed as per immunofluorescence staining. TUNEL staining was performed using an *In Situ* Cell Death Detection Kit, Fluorescein (Roche, Basel, Switzerland), according to the manufacturer's instructions.

### 2.7. Stimulation with LPS

As we wanted to activate CD14-dependent signalling, a low dose of LPS was necessary as it is known that higher concentrations of LPS result in CD14-independent TLR4 signalling [19, 20]. In previous experiments using the mouse epithelial cell line CMT93, we tested different LPS concentrations and time points [11]. In our hands, 0.1 *μ*g/ml and 6 hours were the best concentration and incubation time to measure CD14-dependent cytokine induction. After 10 days of growth, colonic organoids were stimulated with LPS (Sigma-Aldrich, St. Louis, USA) as described previously [11]. In brief, growth media was removed and replaced with 500 *μ*l fresh growth media without Cellshield and containing either 0.1 *μ*g/ml LPS or no LPS as the control. After 6 hours of incubation at 37°C and 5% CO_2_, media was removed and the Matrigel® was dissolved in ice-cold DPBS. Two to 6 wells were pooled, and cells were centrifuged, resuspended in RNA Quick-RNA™ Micro Prep Kit Lysis Buffer (Zymo Research, Irvine, USA), and stored at -80°C for later RNA isolation.

### 2.8. Stimulation with *E. coli* Nissle 1917

An ampicillin-resistant, GFP-expressing *E. coli* Nissle 1917 strain (*Ec*N) was grown at 37°C shaking in lysogeny broth (LB) media containing 100 *μ*g/ml ampicillin until an OD_600_ of 1.0 was reached. As we wanted to compare LPS- and *Ec*N-stimulated organoids, we chose the same incubation time for both treatments. Moreover, in preliminary experiments with our WT organoids, 6 hours of *Ec*N stimulation reproducibly resulted in significant increases in both TNF*α* gene expression and protein production compared to unstimulated cells. The bacterial suspension was diluted 1 : 25 in Cellshield-free organoid growth media. Media of organoids grown for 10 days was removed and replaced with the bacteria-containing growth media and incubated for 6 hours at 37°C and 5% CO_2_. Media was removed, and organoid cells were harvested for RNA isolation according to the LPS stimulation protocol.

### 2.9. RNA Isolation

RNA isolation was performed with RNA Quick-RNA™ Micro Prep Kit (Zymo Research, USA) according to the manufacturer's instructions.

### 2.10. Quantitative Real-Time PCR (qPCR)

For quantification of gene expression after stimulation, up to 1 *μ*g RNA was used for cDNA synthesis using QuantiTect® Reverse Transcription Kit (Qiagen®, Hilden, Germany) according to the manufacturer's instructions. For qPCR analysis, a QuantStudio™ 6 Flex Real-Time PCR System (Applied Biosystems™, Foster City, USA) was used. qPCR was performed using either a TaqMan®-based assay (*Actb*: Mm00607939_s1; *Tnfα*: Mm00443258_m1; *Mki67*: Mm01278617; *LgR5*: Mm00438890_m1; *Smoc2*: Mm00491553_m1; *Clca4b*: Mm01616360_m1; Applied Biosystems®, Foster City, USA) or SYBR™ Green-Based QuantiTect® Primer Assays (*Actb*: Mm_Actb_2_SG; *Cldn4*: Mm_Cldn4_1_SG; *Cldn8*: Mm_Cldn8_1_SG; *Ocln*: Mm_Ocln_1_SG; *Tjp1*: Mm_Tjp1_1_SG; *Cxcl1*: Mx_Cxcl1_1_SG; *Cxcl2*: Mx_Cxcl2_1_SG; *Cxcl5*: Mm_Cxcl5_2_SG; Qiagen®, Hilden, Germany). Each sample was measured in triplicate. *Actb* was used as endogenous reference control gene. Relative gene expression was calculated using the 2^−*Δ*Ct^ method.

### 2.11. Immunoassays

TNF*α* was quantified in the supernatants of LPS- and *Ec*N-stimulated organoids using an ELISA MAX™ Deluxe Set Mouse TNF*α* (BioLegend, San Diego, USA), according to the manufacturer's instructions. Samples and standards were prepared in duplicate and measured at 450 nm with a plate reader (VICTOR™ X3, PerkinElmer, Waltham, MA, USA). CXCL1, CXCL2, and CXCL5 were quantified using a multiplex immunoassay (ProcartaPlex, Life Technologies, Carlsbad, USA) according to the manufacturer's instructions. Concentrations were determined by parallel standard curves for each parameter.

### 2.12. Western Blot Analysis

Organoids were analysed for TJ expression by Western blot analysis after treatment with LPS or *Ec*N. Organoids were homogenized with a tissue homogenisator (Ultra Turrax, IKA®-Werke GmbH & Co. KG). Proteins were extracted and separated by sodium dodecyl sulfate polyacrylamide gel electrophoresis, transferred to a nitrocellulose membrane, and immunoblotted using primary antibodies against occludin, claudin8, zonula occludens-1 (Invitrogen), and claudin4 (Abcam). Immunoblotting for GAPDH (GenScript USA Inc., NJ, USA) was used as an internal control. After incubation with the secondary antibody (donkey anti-rabbit IgG (HRP); Abcam), the membrane was developed with a chemiluminescence solution (Bio-Rad Laboratories, Hercules, California, USA) and images were acquired using a ChemiDoc™ Touch Imaging System (Bio-Rad). Blot analysis was performed via ImageJ software (open source).

### 2.13. Statistical Analysis

All statistical analyses were performed using GraphPad Prism6® software (San Diego, USA). Values represent the means with 95% CI or SEM. All values were normalized to the corresponding unstimulated controls in each independent experiment. Accordingly, unstimulated control values were calculated to a value of 1. When two conditions were compared, unpaired *t*-tests were performed. In other cases, one-way analysis of variances followed by Dunnett's multiple comparison tests were used to compare values of stimulated samples of all three knockout genotypes to WT organoids. In the case of unequal variances, a Brown-Forsythe and Welch ANOVA test was performed. *P* < 0.05 was considered significant. ∗ indicates *P* < 0.05, ∗∗ indicates *P* < 0.01, ∗∗∗ indicates *P* < 0.001, and ∗∗∗∗ indicates *P* < 0.0001.

## 3. Results

### 3.1. Colonic Organoids Expand and Differentiate until Day 10 before They Degrade

**Figure 1:**
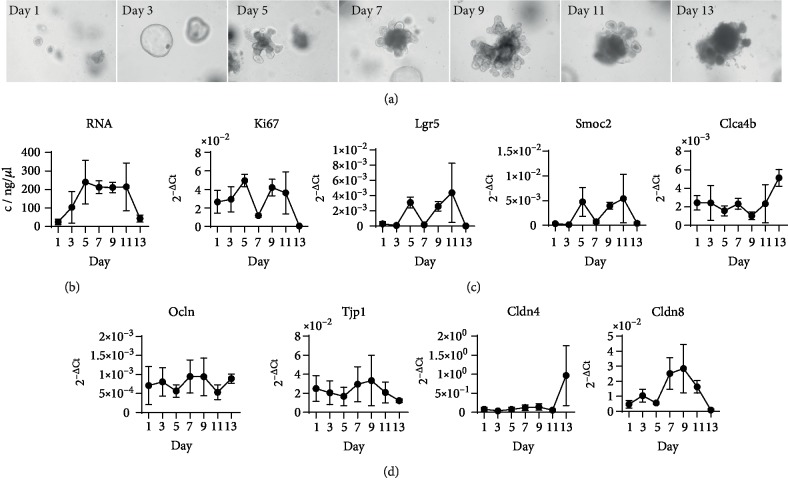
Growth pattern analysis of C57BL/6J colonic organoid culture over time after separation and seeding into new Matrigel®. (a) Representative light phase contrast images. (b) RNA concentrations (*n* = 5). (c) Relative quantification of expression levels of *Ki67*, *Lgr5*, *Smoc2*, and *Clca4b* (*n* = 5). Individual values are represented with the mean and standard deviation. (d) Relative quantification of expression levels of Ocln, Tjp1, Cldn4, and Cldn8 (*n* = 5). Individual values are represented with the mean and standard deviation.

After separation of organoids using a syringe, pictures were taken for growth documentation (Figure 1(a)). Already on day 1, we observed small colonospheres which increased in size until day 3. At day 5, most of the colonospheres started to form crypts around the circular centre, indicating a colonoid state. Dead cells shed into the lumen. On day 7, only minor changes were observed compared to day 5. On day 9, colonic organoids were evenly distributed and developed in the Matrigel®. Starting on day 11, organoids began to break open, resulting in dead cells and debris located outside the colonic organoids. Finally, on day 13, most organoids were disintegrated. At each time point, RNA was isolated from organoids for a later gene expression analysis. RNA concentrations increased until day 5 and remained stable until day 11, after which they rapidly decreased until day 13.

To characterise cell proliferation within organoids, *Ki67* expression was determined on the mRNA level by qPCR. An increase in *Ki67* gene expression occurred up to day 5 with a drop in expression at day 7. After a media change on day 7, *Ki67* expression increased and kept increasing until another media change on day 10, after which expression became quite variable. This resulted in widely distributed expression values on day 11. On day 13, Ki67 mRNA was no longer detectable in any organoids. Gene expression of the IEC genes *Smoc2* and *LgR5* was analysed and showed a similar expression pattern as *Ki67*. In contrast, gene expression of the proliferation inhibitor and epithelial differentiation marker *Clca4b* [21] was low and mostly stable up to day 9, after which expression increased until day 13. Furthermore, gene expression of TJ components such as *Ocln*, *Tjp1*, *Cldn4*, and *Cldn8* was analysed. Similar expression levels were detectable between day 1 and day 13 for *Ocln* and *Tjp1*, while *Cldn4* increased and *Cldn8* decreased rapidly on day 13 (Figure 1(d)). Thus, we decided to perform all further experiments using organoids after 10 days of growth, where the morphology showed crypts and the proliferation level was still high.

### 3.2. Colonic Organoids Represent Fully Differentiated Colon Epithelium

**Figure 2:**
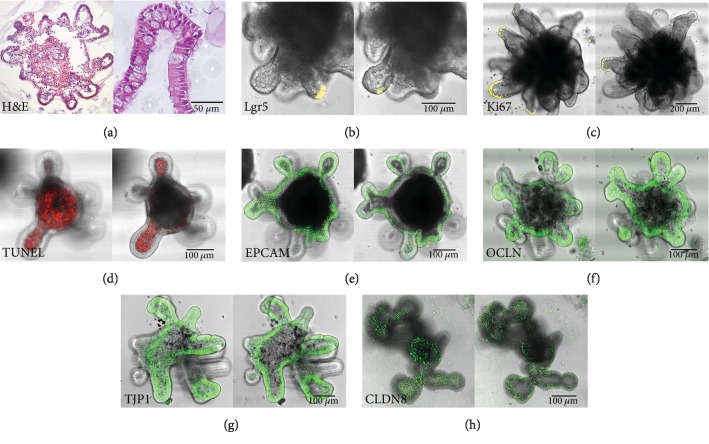
Staining of 10-day-old colonic organoids. (a) Hematoxylin and eosin staining. (b) Lgr5 of *Lgr5-eGFP^+/-^* mice. (c) Immunohistological staining of Ki67. (d–g) Immunofluorescence staining for (d) TUNEL, (e) epithelial cell adhesion molecule, (f) occludin, (g) tight junction protein 1, and (h) claudin-8.

We performed H&E staining on 10-day-old organoid sections to analyse the differentiation and growth status of the IECs (Figure 2(a)). Around the circular lumen, a fully differentiated epithelium with crypts formed, consisting primarily of enterocytes and mucus-containing goblet cells. A few LGR5-positive cells (Figure 2(b)) were present at the base of crypts, whereas high amounts of Ki67-positive cells (Figure 2(c)) were located at the base and sides of crypt and were not found elsewhere in the organoids. Dead cells collected in the lumen as confirmed by whole mount TUNEL staining (Figure 2(d)). The organoid cell layer stained positive for epithelial cell adhesion molecule (EPCAM or CD326) which confirmed these cells as epithelial cells (Figure 2(e)). These cells were also positive for the intracellular TJ proteins occludin (OCLN) (Figure 2(f)), tight junction protein 1 (TJP1) (Figure 2(g)), and claudin-8 (CLDN8) (Figure 2(h)).

### 3.3. Decreased Expression of Tight Junction Components and Proinflammatory Response in *Cd14*^−/−^ Epithelial Cells upon LPS Stimulation

**Figure 3:**
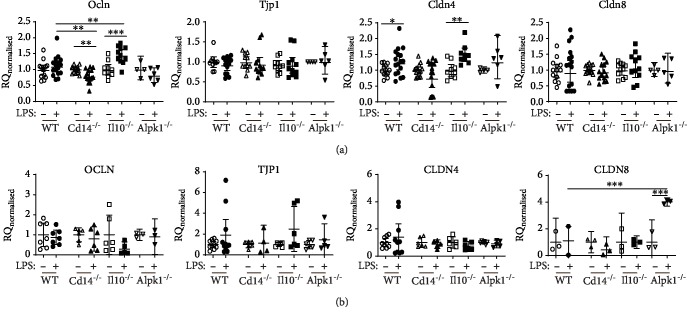
Expression of tight junction after LPS stimulation. Organoids of different genotypes were stimulated with LPS for 6 hours. Change in tight junction component gene and protein expression was analysed and compared to wild type (WT). Values were normalized to unstimulated controls within each genotype in each experiment and represented the means with 95% CI and SEM. Unstimulated: WT *n* = 3‐13, Cd14^−/−^*n* = 3‐14, Il10^−/−^*n* = 3‐9, and Alpk1^−/−^*n* = 3‐6. Stimulated: WT *n* = 2‐17, Cd14^−/−^*n* = 3‐15, Il10^−/−^*n* = 3‐11, and Alpk1^−/−^*n* = 3‐6. (a) Gene expression. RQ = relative quantification. (b) Detection of TJ proteins by Western blot analysis of lysates from organoids after 6 h of LPS stimulation. Densitometric quantification normalized to GAPDH was calculated as change from the unstimulated control. RD = relative density %. Stimulated versus unstimulated values were compared using unpaired *t*-tests. Welch's correction was used in case of unequal variances. One-way analysis of variance followed by Dunnett's multiple comparisons was performed. Each knockout genotype was compared to WT. ^∗∗^*P* < 0.01, ^∗∗∗^*P* < 0.001.

**Figure 4:**
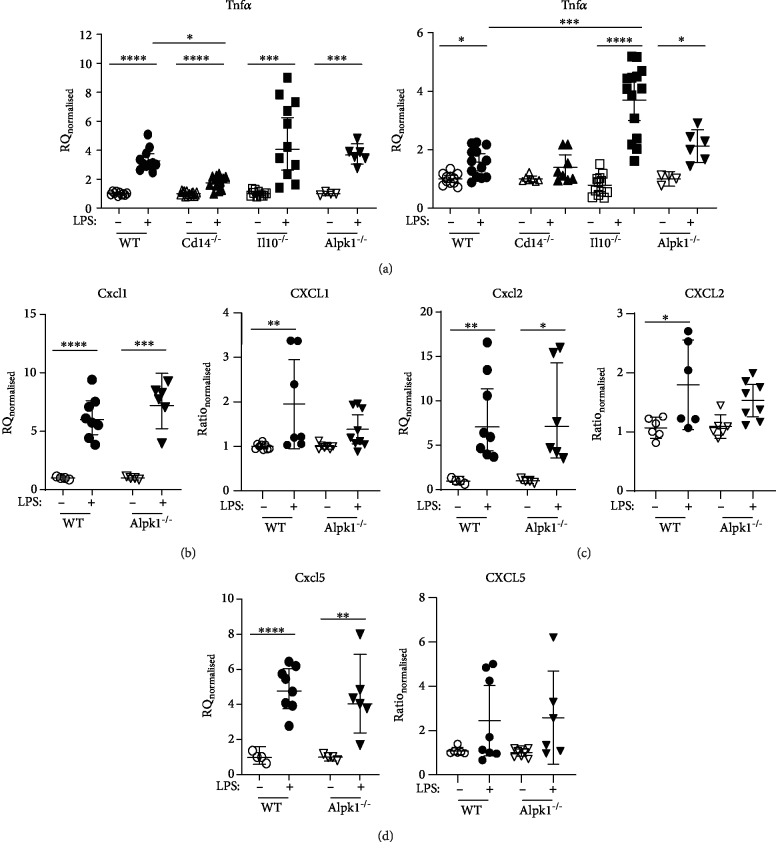
Expression of proinflammatory mediators after LPS stimulation. Changes in gene expression of cytokines and their supernatant concentration from organoids stimulated with LPS for 6 hours were analysed. Knockout organoids were compared to wild type (WT). Values are normalized to unstimulated controls within each genotype for each experiment and represent the means with 95% CI. Unstimulated: WT *n* = 4‐10, *Il10*^−/−^*n* = 11, *Cd14*^−/−^*n* = 7‐14, and *Alpk1*^−/−^*n* = 4‐8. Stimulated: WT *n* = 9‐13, *Il10*^−/−^*n* = 11‐14, *Cd14*^−/−^*n* = 8‐13, and *Alpk1*^−/−^*n* = 6‐9. (a) *Tnfα*/TNF*α*, (b) *Cxcl1*/CXCL1, (c) *Cxcl2*/CXCL2, and (d) *Cxcl5*/CXCL5. Stimulated versus unstimulated values were compared using unpaired *t*-tests. In the case of unequal variances, Brown-Forsythe and Welch ANOVA test was performed. In all other cases, a one-way analysis of variance followed by Dunnett's multiple comparisons was performed. Each knockout genotype was compared to WT. ^∗^*P* < 0.05, ^∗∗^*P* < 0.01, ^∗∗∗^*P* < 0.001, and ^∗∗∗∗^*P* < 0.0001.

After 6 hours of LPS stimulation, RNA or protein samples were taken from the organoids and expression levels of different genes involved in tight junctions (*Ocln*, *Tjp1*, *Cldn4*, and *Cldn8*) (Figure 3 and Supplemental [Supplementary-material supplementary-material-1]) and immune response (*Tnfα*, *Cxcl1*, *Cxcl2*, and *Cxcl5*) were analysed (Figure 4). To adjust batch effects, all values were normalized to the corresponding unstimulated controls in each independent experiment. Compared to WT organoids, *Ocln* expression significantly increased in *Il10*^−/−^ organoids after exposure to LPS but was significantly decreased in *Cd14*^−/−^ organoids after LPS stimulation (Figure 3(a)). Western blot analysis of OCLN showed no differences in all organoids after 6 h of LPS stimulation (Figure 3(b)). Gene and protein expression of *Tjp1* was not altered in any of the organoids after LPS challenge (Figures 3(a) and 3(b)). LPS exposure significantly increased *Cldn4* expression in WT and *Il10*^−/−^ and slightly in *Alpk1*^−/−^ organoids (Figure 3(a)). In *Cd14*^−/−^ organoids, *Cldn4* expression decreased slightly upon LPS exposure. These differences were not detectable on protein level (Figure 3(b)). As for *Cldn8*, expression levels remained unchanged for all genotypes (Figure 3(a)); however, CLDN8 expression was increased in *Alpk1*^−/−^ organoids after LPS stimulation (Figure 3(b)).

Upon LPS stimulation, gene expression of proinflammatory *Tnfα* was increased in all organoids, but this increase was significantly less in *Cd14*^−/−^ organoids compared to WT organoids (Figure 4(a)). Protein levels of TNFa proteins also increased in all organoids especially in *Il10^−/−^* epithelial cells. In contrast, TNF*α* secretion was not increased in *Cd14*^−/−^ epithelial cells after LPS stimulation (Figure 4(a)). Chemokines *Cxcl1*, *Cxcl2*, and *Cxcl5* were increased after LPS stimulation in both investigated organoid genotypes, WT and *Alpk1*^−/−^ (Figures 4(b)–4(d)). However, multiplex analysis of CXCL1, CXCL2, and CXCL5 revealed only an increase in WT organoids. *Alpk1*^−/−^ epithelial cells showed only a modest increase in chemokine expression. Thus, LPS triggers a proinflammatory effect in all observed organoids but had lower effects in *Cd14*^−/−^ and *Alpk1*^−/−^ epithelial cells. Additionally, expression of TJ components *Ocln* and *Cldn4* is affected by LPS in *Cd14^−/−^* organoids and CLDN8 seems to be altered in *Alpk1^−/−^* epithelial cells.

### 3.4. *Alpk1* Deficiency Affects Tight Junction Component Expression after *E. coli* Nissle 1917 Infection of Colonic Organoids

**Figure 5:**
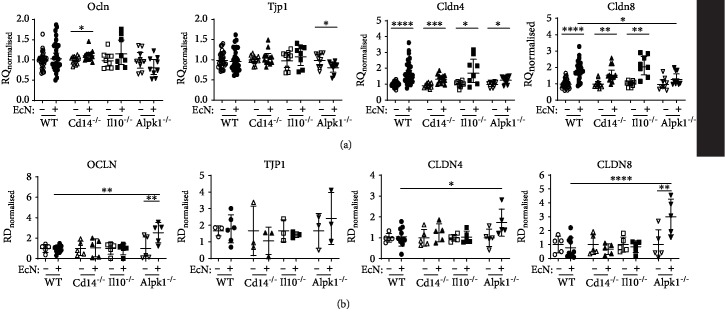
Expression of tight junction genes after *E. coli* Nissle stimulation. Wild-type (WT) and knockout organoids were stimulated with *E. coli* Nissle 1917 for 6 hours and analysed for changes in tight junction component gene and protein expression. Differences between knockout and WT organoids were investigated. Values were normalized to unstimulated controls within each genotype for each experiment and represent the means with 95% CI. Unstimulated: WT *n* = 3‐30, *Cd14*^−/−^*n* = 3‐11, *Il10*^−/−^*n* = 3‐9, and *Alpk1*^−/−^*n* = 3‐9. Stimulated: WT *n* = 6‐29, *Cd14*^−/−^*n* = 3‐12, *Il10*^−/−^*n* = 3‐8, and *Alpk1*^−/−^*n* = 3‐9. (a) Gene expression. RQ = relative quantification. (b) Detection of TJ proteins by Western blot analysis of lysates from organoids after 6 h of *Ec*N stimulation. Densitometric quantification normalized to GAPDH was calculated as change from the unstimulated control. RD = relative density %. Stimulated versus unstimulated values were compared using unpaired *t*-tests. Welch's correction was used in case of unequal variances. One-way analysis of variance followed by Dunnett's multiple comparisons was performed. Each knockout genotype was compared to WT. ^∗^*P* < 0.05, ^∗∗^*P* < 0.01, ^∗∗∗^*P* < 0.001, and ^∗∗∗∗^*P* < 0.0001.

**Figure 6:**
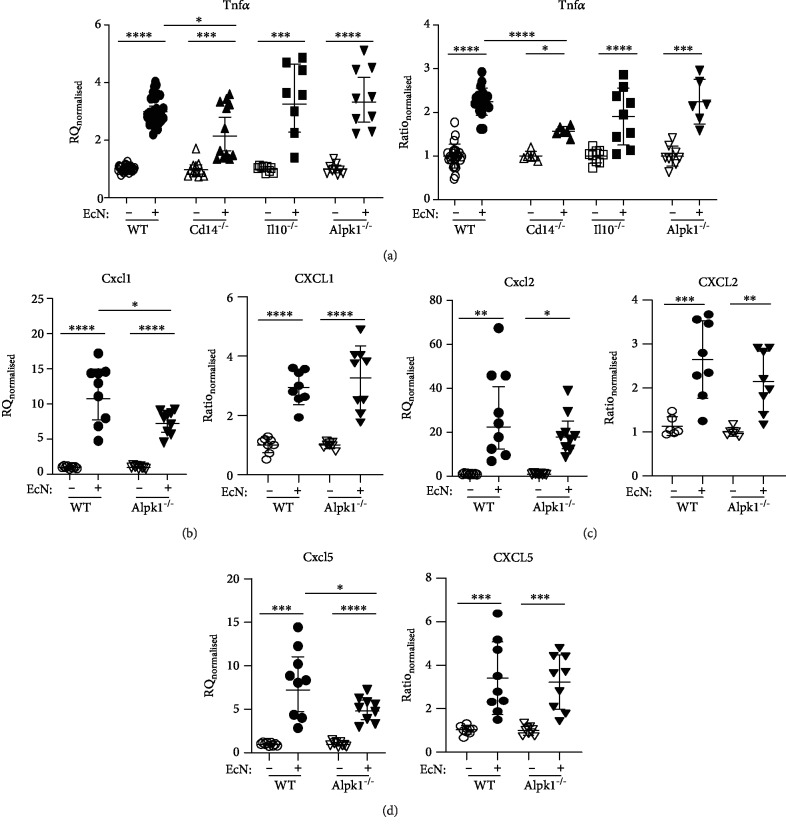
Expression of proinflammatory mediators after *E. coli* Nissle stimulation. Changes in gene expression of cytokines and their supernatant concentration after 6 hours of *E. coli* Nissle 1917 (*Ec*N) stimulation were compared between wild-type (WT) and knockout organoids. Values were normalized to unstimulated controls within each genotype for each experiment and represent the means with 95% CI. Unstimulated: WT *n* = 6‐30, *Cd14*^−/−^*n* = 6‐11, *Il10*^−/−^*n* = 9, and *Alpk1*^−/−^*n* = 9. Stimulated: WT *n* = 6‐27, *Il10*^−/−^*n* = 8‐9, *Cd14*^−/−^*n* = 6‐12, and *Alpk1*^−/−^*n* = 6‐9. (a) *Tnfα*/TNF*α*, (b) *Cxcl1*/CXCL1, (c) *Cxcl2*/CXCL2, and (d) *Cxcl5*/CXCL5. Stimulated versus unstimulated values were compared using unpaired *t*-tests. Welch's correction was used in case of unequal variances. One-way analysis of variance followed by Dunnett's multiple comparisons was performed. Each knockout genotype was compared to WT. ^∗^*P* < 0.05, ^∗∗^*P* < 0.01, ^∗∗∗^*P* < 0.001, and ^∗∗∗∗^*P* < 0.0001.

After 6 hours of *Ec*N stimulation, gene and protein expression of tight junctions and inflammatory cytokines was measured (Figures 5 and 6 and Supplemental [Supplementary-material supplementary-material-1]). WT and *Il10*^−/−^ organoids showed no changes in *Ocln* and *Tjp1* expression compared to uninfected controls (Figures 5(a) and 5(b)); *Ocln* expression was significantly increased in *Cd14*^−/−^ organoids (Figure 5(a)). Upon *Ec*N infection, *Alpk1*^−/−^ organoids had slight but not statistically significant decreased *Ocln* expression (Figure 5(a)). However, Western blot analysis showed an increased OCLN level compared to WT organoids (Figure 5(b)).

Significantly reduced *Tjp1* expression was measured in *Alpk1*^−/−^ epithelial cell, but no differences were detectable in TJP1 levels in all genotypes (Figures 5(a) and 5(b)). *Cldn4* expression significantly increased upon *Ec*N exposure in all genotypes although only increased CLDN4 expression was determined in *Alpk1*^−/−^ organoids (Figures 5(a) and 5(b)). *Cldn8* expression was significantly elevated in WT, *Il10*^−/−^, and *Cd14*^−/−^ organoids upon *Ec*N stimulation but notably not in *Alpk1*^−/−^ organoids (Figures 5(a) and 5(b)). However, increased protein expression was measured in *Alpk1*^−/−^ epithelial cell (Figure 5(b)).

After *Ec*N stimulation, *Tnfα*/TNF*α* expression was elevated for all organoids, but this increase was significantly lower in *Cd14*^−/−^ compared to WT organoids (Figure 6(a)). Expression of *Cxcl1*, *Cxcl2*, and *Cxcl5* was increased upon *Ec*N exposure in WT and *Alpk1*^−/−^ organoids, but the increases in *Cxcl1* and *Cxcl5* expression were significantly lower in *Alpk1*^−/−^ organoids compared to WT organoids (Figures 6(b)–6(d)).

No difference in chemokine secretion between *Alpk1*^−/−^ organoids and WT epithelial cells was observed in multiplex analysis of supernatants (Figures 6(b)–6(d)).

Together, our data show (i) decreased *Ocln* and *Tnfα* gene expression in LPS-stimulated *Cd14*^−/−^ organoids compared to WT epithelial cells; (ii) increased CLDN8 protein expression after LPS stimulation in *Alpk1*^−/−^ cells; (iii) decreased *Cldn8*, *Cxcl1*, and *Cxcl5* gene expression and increased protein expression of TJ components such as CLDN4, CLDN8, and OCLN in *Ec*N-stimulated *Alpk1*^−/−^ organoids compared to stimulated WT organoids; and (iv) less *Tnfα*/TNF*α* production in *Cd14*^−/−^ cells compared to WT epithelial cells. In conclusion, these data indicate that *Cd14*, but not *Alpk1*, alters the response to LPS stimulation in colonic epithelial cells, whereas *Alpk1* is more involved in the proinflammatory response to bacterial challenge.

## 4. Discussion

In the present study, we utilized colon-derived 3D organoids derived from WT, *Il10*^*-/*-^, *Cd14^−/−^*, and *Alpk1^−/−^* knockout mice to investigate the response to either purified LPS or whole Gram-negative bacteria. We show for the first time a role for ALPK1 in TJ component regulation after infection with the Gram-negative bacteria *Ec*N. In addition, our results corroborate the role of CD14 in LPS-triggered proinflammatory and TJ component responses [11]. Although there are reports of *Il10* being expressed in IECs [22], IL10 itself is primarily produced by immune cells where it functions to regulate the intestinal homeostasis and barrier integrity via its anti-inflammatory effects [23, 24]. In our study, the absence of IL10 did not alter the response of colonic organoids to *Ec*N compared to WT cells.

There are several variations described to culture, separate, and passage 3D organoids which result in different periods of growth and differentiation [18, 25, 26]. Our isolated crypt stem cells developed into colonospheres within 24 hours. The observed growth correlated with an increase in RNA concentration, proliferation, and expression of stem cell genes in the first 5 days. Organoid growth and differentiation are highly dependent on the supplied media components. We assume that the drop in *Ki67*, *Lgr5*, and *Smoc2* expression after day 5 resulted from consumption of the differentiation inhibitor Y-27632. After a media exchange, organoids proliferated once more and formed more crypts. Eleven days after seeding, organoids began to break open, and by day 13, they were almost uniformly disrupted. In another study by Thalheim et al. [27] investigating the growth pattern of intestinal organoids, an equilibrium in the different epithelial cell type fractions was reached after 10 days. When we observed our organoids after 10 days, LGR5- and KI67-positive IECs were at the base of crypts indicating proliferating stem cell populations. Both enterocyte and mucus-producing goblet cell population were observed by H&E staining, immunofluorescence, and cellular junction staining, and intestinal desquamation and cell turnover were visible. Therefore, we chose day 10 to begin our stimulation and infection experiments since at this time point colonic organoids were viable, differentiated, and demonstrated mature cellular junctions and crypt-villus structure.

IEC is primarily exposed to bacteria and LPS on their apical surface; however, the basolateral surface is also exposed when the barrier is compromised or upon infection with invasive pathogens. Several studies have observed an effect of physiological concentrations of basally and apically applied LPS on IEC TJ permeability [28–30]. The way of infection in 3D organoids is dependent on the regarded strain [31]. In a study of Sabharwal et al. in 2016, polarized T84 cells were infected with *Ec*N [32]. Apical *Ec*N infection triggered only a slight upregulation of *Cxcl1* and an additional downregulation of Il8 expression. When infecting from the basolateral side, *Ec*N greatly enhanced expression of *Il8* and *Cxcl1* [32]. In our organoid system, stimulation and infection is performed on the basolateral surface as *Ec*N infection triggers epithelial activation at this site.

LPS is a pathogen-associated molecular pattern that is recognized by TLR4/CD14. Signalling via TLR4/CD14 leads to NF-*κ*B activation and subsequent proinflammatory gene expression [33]. In our study, we used a LPS concentration of 0.1 *μ*g/ml, which was previously shown to induce an epithelial response *in vitro* [11]. After 6 hours of LPS stimulation or *Ec*N infection, all organoid lines (WT, *Cd14*^−/−^, *Il10*^−/−^, and *Alpk1*^−/−^) had increased expression of *Tnfα*, proving a positive reaction to the stimulus. However, the increase in *Tnfα*/TNF*α* expression in the *Cd14^−/−^* organoids was lower compared to WT organoids, validating the role of CD14 in LPS-triggered proinflammatory signalling [20].

ALPK1 does not recognize LPS itself but rather a metabolic intermediate involved in LPS biosynthesis. Accordingly, our results confirmed that the lack of ALPK1 barely affects IEC proinflammatory response to LPS exposure alone, as TNF*α* expression was similar compared to LPS-stimulated WT cells. Upon bacterial infection of IECs, ALPK1 has been shown to signal through TRIF/TRAF6 to NF-*κ*B, triggering secretion of proinflammatory cytokines like IL8 and TNF*α* [34]. However, upregulation of chemokine *Cxcl1* and *Cxcl5* was significantly less in *Alpk1*^−/−^ organoids infected with *Ec*N compared to WT organoids, but an *Alpk1* knockout did not result in decreased levels of proinflammatory mediators in the supernatants upon bacterial stimulation of colonic organoids. Therefore, in our experimental setup, the lack of ALPK1 did not result in decreased levels of proinflammatory mediators suggesting a minor role of the ALPK1 signalling pathway in epithelial cells.

Upon LPS stimulation of WT organoids, we did not observe changes in the expression of TJ component genes *Ocln*, *Tjp1*, or *Cldn8* but did record a significant increase in *Cldn4* expression. *Ec*N infection of WT organoids increased *Cldn4* and *Cldn8* expression, suggesting a positive effect of these bacteria on the IEC barrier. Protein levels of occludin showed no differences in WT, *Il10*^−/−^, and *Cd14^−/−^* organoids suggesting a delay in TJ regulation. However, *Ocln* expression was significantly reduced in *Cd14^−/−^* organoids upon LPS exposure. Furthermore, *Cd14^−/−^* organoids tend to have lower expression levels of *Cldn4* upon LPS stimulation. As occludin plays an important role in the stability of tight junctions, these gene expression data support earlier findings of our group showing a protective role for CD14 in the intestinal barrier function [11]. A similar decrease in TJ component gene expression was not seen in *Cd14*^−/−^ organoids with *Ec*N infection, suggesting possible contextual differences in LPS presentation to TLR4/CD14. As we have shown *in vivo*, epithelial-immune cell crosstalk could be necessary for CD14-dependent impairment of the intestinal barrier upon *Ec*N infection [12, 35]. Alternatively, other PRRs like TLR2 may be compensating for the lack of CD14 [36].

In addition, infection with whole Gram-negative bacteria revealed a significant increase in all analysed TJ proteins in *Alpk1*^−/−^ organoids, while RNA expression levels of Tjp1 and Cldn8 were decreased. These results indicate that ALPK1 plays an important role regulating tight junction component expression in IECs which could, in turn, lead to an altered IEC barrier. In contrast, ALPK1 did not affect the nonhematopoietic compartment in a *H. hepaticus*-induced colitis mouse model. Rather, ALPK1 influenced the regulation of IL12 production in bone marrow-derived macrophages [13].

Our group previously described *Alpk1* as a *Cdcs1* locus candidate gene for IL10 deficiency-induced colitis severity in mice [4] and recently excluded a T cell-dependent role for ALPK1 in colitis progression [5]. Our present study shows an influence of *Alpk1* on epithelial barrier components in response to *Ec*N and therefore perhaps to tolerance to commensal bacteria. A defect in *Alpk1* could therefore contribute to a disruption of barrier function upon bacterial infection and be a cause for exacerbation of colitogenic inflammation. Future studies concerning IBD and intestinal barrier integrity should therefore investigate whether *Alpk1*-dependent signalling is involved in disease progression.

## Data Availability

The raw data supporting the conclusions of this manuscript are available from the corresponding author on reasonable request to any qualified researcher.

## Supplementary Materials

Supplementary Materials Supplemental Figure 1: detection of TJ proteins by Western blot analysis of lysates from organoids after 6 h of LPS stimulation (A) and EcN stimulation (B). (A) Samples were loaded in a random order as follows from left to right: membrane 1: WT control, Il10^−/−^+LPS, WT+LPS, Il10^−/−^ control, Cd14^−/−^ control, Il10^−/−^ control, Cd14^−/−^ control, Cd14^−/−^ control, Il10^−/−^ control; membrane 2: WT+LPS, WT+LPS, WT control, WT control, Cd14^−/−^+LPS, Il10^−/−^+LPS, Il10^−/−^+LPS, Cd14^−/−^+LPS, Cd14^−/−^+LPS; membrane 3: occludin: WT control, WT LPS, Cd14^−/−^ control, Cd14^−/−^+LPS, Il10^−/−^ control, Il10^−/−^+LPS, Alpk1^−/−^ control, Alpk1^−/−^+LPS, WT+LPS; TJP1, claudin 4, claudin 8, and GAPDH: Alpk1^−/−^ control, Alpk1^−/−^control, Alpk1^−/−^ control, Alpk1^−/−^+LPS, Alpk1^−/−^+LPS, Alpk1^−/−^+LPS. (B) Samples were loaded as follows from left to right: WT control, WT+EcN, Cd14^−/−^ control, Cd14^−/−^+EcN, Il10^−/−^ control, Il10^−/−^+EcN, Alpk1^−/−^ control, Alpk1^−/−^+EcN, WT+EcN.
