# Supplementary material for: CD14 and ALPK1 Affect Expression of Tight Junction Components and Proinflammatory Mediators upon Bacterial Stimulation in a Colonic 3D Organoid Model
Source: Stem Cells Int. 2020 Feb 1;2020:4069354. doi: 10.1155/2020/4069354 (PMC7016478; doi:10.1155/2020/4069354)
Supplement: Supplementary Materials — Supplemental Figure 1: detection of TJ proteins by Western blot analysis of lysates from organoids after 6 h of LPS stimulation (A) and EcN stimulation (B). (A) Samples were loaded in a random order as follows from left to right: membrane 1: WT control, Il10−/−+LPS, WT+LPS, Il10−/− control, Cd14−/− control, Il10−/− control, Cd14−/− control, Cd14−/− control, Il10−/− control; membrane 2: WT+LPS, WT+LPS, WT control, WT control, Cd14−/−+LPS, Il10−/−+LPS, Il10−/−+LPS, Cd14−/−+LPS, Cd14−/−+LPS; membrane 3: occludin: WT control, WT LPS, Cd14−/− control, Cd14−/−+LPS, Il10−/− control, Il10−/−+LPS, Alpk1−/− control, Alpk1−/−+LPS, WT+LPS; TJP1, claudin 4, claudin 8, and GAPDH: Alpk1−/− control, Alpk1−/−control, Alpk1−/− control, Alpk1−/−+LPS, Alpk1−/−+LPS, Alpk1−/−+LPS. (B) Samples were loaded as follows from left to right: WT control, WT+EcN, Cd14−/− control, Cd14−/−+EcN, Il10−/− control, Il10−/−+EcN, Alpk1−/− control, Alpk1−/−+EcN, WT+EcN. [file 4069354.f1.docx]

**Supplemental information**

**CD14 and ALPK1 affect expression of tight junction components and pro-inflammatory mediators upon bacterial stimulation in a colonic 3D organoid model**

Pascal Brooks^1^*; Talke zur Bruegge^1^*; Erin C. Boyle^1^; Stefan Kalies^2,3^; Santiago Nahuel Villarreal^1^; Andrea Liese^1^; André Bleich^1#^; Manuela Buettner^1#^


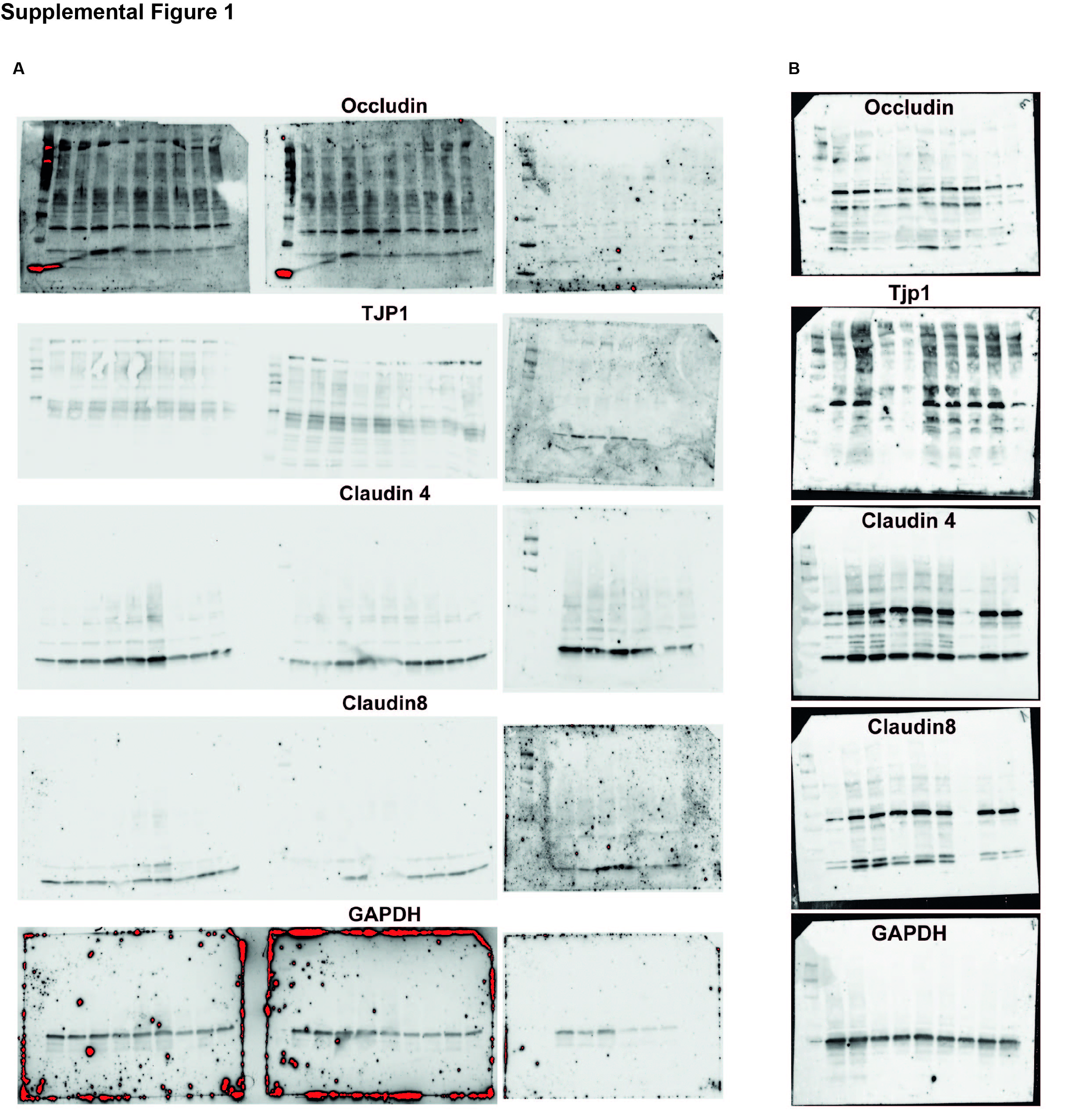


Supplemental Figure 1: Detection of TJ proteins by Western blot analysis of lysates from organoids after 6 h of LPS stimulation (A) and EcN stimulation (B).

(A) Samples were loaded in a random order as follows from left to right: Membrane 1: WT control, *Il10^-/-^* + LPS, WT + LPS, *Il10^-/-^* control, *Cd14^-/-^* control, *Il10^-/-^* control, *Cd14^-/-^* control, *Cd14^-/-^* control, *Il10^-/-^* control; Membrane 2: WT + LPS, WT + LPS, WT control, WT control, *Cd14^-/-^* + LPS, *Il10^-/-^* + LPS, *Il10^-/-^* + LPS, *Cd14^-/-^* + LPS, *Cd14^-/-^* + LPS; Membrane 3: Occludin: WT control, WT LPS, *Cd14^-/-^* control, *Cd14^-/-^* + LPS, *Il10^-/-^* control, *Il10^-/-^* + LPS, *Alpk1^-/-^* control, *Alpk1^-/-^* + LPS, WT + LPS; TJP1, claudin 4, claudin 8 and GAPDH: *Alpk1^-/-^* control, *Alpk1^-/-^* control, *Alpk1^-/-^* control, *Alpk1^-/-^* + LPS, *Alpk1^-/-^* + LPS, *Alpk1^-/-^* + LPS

(B) Samples were loaded as follows from left to right: WT control, WT + *Ec*N, *Cd14^-/-^* control, *Cd14^-/-^* + *Ec*N, *Il10^-/-^* control, *Il10^-/-^* + *Ec*N, *Alpk1^-/-^* control, *Alpk1^-/-^* + *Ec*N, WT + *Ec*N
